# Supplementary material for: Presepsin cut-off value for diagnosis of sepsis in patients with renal dysfunction
Source: PLoS One. 2022 Sep 14;17(9):e0273930. doi: 10.1371/journal.pone.0273930 (PMC9473387; doi:10.1371/journal.pone.0273930)
Supplement: Attachment — Submitted filename: Response for reviewers.docx [file pone.0273930.s002.docx]

Response to Reviewers (Academic editor)

Journal Requirements:

When submitting your revision, we need you to address these additional requirements.

1. Please ensure that your manuscript meets PLOS ONE's style requirements, including those for file naming. The PLOS ONE style templates can be found at
   <https://journals.plos.org/plosone/s/file?id=wjVg/PLOSOne_formatting_sample_main_body.pdf> and 
   <https://journals.plos.org/plosone/s/file?id=ba62/PLOSOne_formatting_sample_title_authors_affiliations.pdf>

Response

Our manuscript now meets PLOS ONE's style requirements.

2. Please provide additional details regarding participant consent. In the ethics statement in the Methods and online submission information, please ensure that you have specified (1) whether consent was informed and (2) what type you obtained (for instance, written or verbal, and if verbal, how it was documented and witnessed). If your study included minors, state whether you obtained consent from parents or guardians. If the need for consent was waived by the ethics committee, please include this information.

If you are reporting a retrospective study of medical records or archived samples, please ensure that you have discussed whether all data were fully anonymized before you accessed them and/or whether the IRB or ethics committee waived the requirement for informed consent. If patients provided informed written consent to have data from their medical records used in research, please include this information

Once you have amended this/these statement(s) in the Methods section of the manuscript, please add the same text to the “Ethics Statement” field of the submission form (via “Edit Submission”).

For additional information about PLOS ONE ethical requirements for human subjects research, please refer to <http://journals.plos.org/plosone/s/submission-guidelines#loc-human-subjects-research>.

Response

We have now provided additional details regarding participant consent in the Methods (lines 60–69). Furthermore, we added the same text to the “Ethics Statement” field of the submission form.

3. In your Data Availability statement, you have not specified where the minimal data set underlying the results described in your manuscript can be found. PLOS defines a study's minimal data set as the underlying data used to reach the conclusions drawn in the manuscript and any additional data required to replicate the reported study findings in their entirety. All PLOS journals require that the minimal data set be made fully available. For more information about our data policy, please see <http://journals.plos.org/plosone/s/data-availability>.

"Upon re-submitting your revised manuscript, please upload your study’s minimal underlying data set as either Supporting Information files or to a stable, public repository and include the relevant URLs, DOIs, or accession numbers within your revised cover letter. For a list of acceptable repositories, please see <http://journals.plos.org/plosone/s/data-availability#loc-recommended-repositories>. Any potentially identifying patient information must be fully anonymized.

Important: If there are ethical or legal restrictions to sharing your data publicly, please explain these restrictions in detail. Please see our guidelines for more information on what we consider unacceptable restrictions to publicly sharing data: <http://journals.plos.org/plosone/s/data-availability#loc-unacceptable-data-access-restrictions>. Note that it is not acceptable for the authors to be the sole named individuals responsible for ensuring data access.

We will update your Data Availability statement to reflect the information you provide in your cover letter.

Response

We have uploaded the study’s minimal underlying data set as Supporting Information files.

Response to Reviewer #1

Major comments

1. In lines 45-47, it is not clear how were the enrolled patients selected to measure presepsin levels. Were they suspected to have sepsis or infection? Were they positive for some of the diagnostic criteria of the sepsis-1, sepsis-3 or Sep-C? The inclusion criteria were not clear.

Response

Thank you for your question. We included patients whose serum presepsin levels were measured at our hospital from August 1, 2017, to July 31, 2018, and who had records of eGFR, blood pressure, pulse, and body temperature on the same day. We presumed that these patients had symptoms of infectious disease, such as fever, and that it was necessary to distinguish sepsis. We added these explanations in lines 49–50.

2. The authors employed sepsis-1, sepsis-3 or Sep-C as gold standards, presepsin will not exceed the diagnostic values of these diagnostic criteria. The authors should set more valuable clinical outcomes for gold standard, e.g. prognosis of the patients. In addition, the sepsis-1, sepsis-3 or Sep-C were defined for the quick diagnosis for sepsis, the measurement of presepsin may not contribute the fast and easy diagnosis of sepsis.

Response

Thank you for your advice. Presepsin was discovered as a useful marker for the early diagnosis of sepsis and has been reported to also be associated with prognosis. Therefore, it is meaningful to analyze its relationship with prognosis. However, we planned this study to respond to the following questions. How well can presepsin predict the diagnosis of sepsis? Does the diagnostic accuracy of presepsin differ depending on the degree of renal dysfunction? Furthermore, we aimed to explore the cutoff value of presepsin for the diagnosis of sepsis in patients with renal impairment.

Regarding prognosis, we analyzed the relationship between death and readmission for 6 months after presepsin measurement, as far as we could confirm from the medical records. The median serum presepsin level was significantly higher in the death group (N = 125) than in the surviving group (N = 470) (876 vs. 446 pg/mL, p < 0.01 by the Mann-Whitney U test). However, the AUC in the ROC analysis was as low as 0.67 ± 0.03. In addition, the median serum presepsin level was not significantly different between the readmission (N = 194) and non-readmission groups (N = 401) (597 vs. 503 pg/mL). From these analyses, it cannot be concluded that the prognostic ability of serum presepsin is high.

3. In line 110-111, why were SOFA and qSOFA not available? Were the data missing? How did the authors analyze the missing data?

Response

We thank you for your question and apologize for our poor explanation. As a result of our retrospective survey of the medical records, there were non-ICU patients who could not be diagnosed with sepsis based on the qSOFA score because there was no record of respiratory rate or change of consciousness. In addition, some ICU patients could not be diagnosed with sepsis by the SOFA score because there was no record of PaO2/FiO2, platelet count, bilirubin level, or Glasgow Coma Scale. We added these explanations in lines 121–129.

4. In Fig 1, eGFR of some of the patients was extremely high ranging from 150-400 mL/min.

Response

Thank you for your comment. Certainly, some of the patients had extremely high eGFRs. It is expected that some of these patients have low muscle mass and overestimated eGFRs. First, we excluded seven patients with extremely high eGFRs (≥ 200) and one pregnant woman. The top 2.5% of the eGFR distribution in all patients was 139.4 pg/mL, and thus we investigated 13 patients with ≥140 pg/mL and excluded nine patients with a body mass index of < 20. This has now been described in the Material and Methods (in lines 54–59).

5. In Fig 5, cut-off values are indicated in the legends, they should be indicated in the figure.

Response

Thank you for your suggestion. We have now indicated the cut-off values in Fig. 5.

6. The authors should make clear the benefit of the measurement of presepsin in the clinical setting.

Response

Thank you for your suggestion. This study included the latest diagnostic criterion, Sep-3, and found that presepsin is inversely proportional to eGFR but that there is no difference in the diagnostic accuracy between eGFR <60 and ≥60 We added the next sentences to the conclusion.

“Our findings indicate that presepsin is useful for diagnosing sepsis regardless of the degree of renal dysfunction by using different cut-offs. We assumed that the optimal cut-offs for the patients in this study were 500 pg/mL for ≥60 and 1000 pg/mL for <60. Future prospective diagnostic studies on Sep-3 are needed to determine the cut-offs for patients with renal dysfunction.”

Response to Reviewer #2

Reviewer #2: Major points

The authors investigated the cutoff value of presepsin for detecting sepsis in patients with renal insufficiency. Although the results were meaningful, there were several problems. Especially, I think that it is difficult to determine the cutoff values of presepsin levels from this study design because of single center study. However, they described that 500 and 1500 pg/mL has been proposed as a cutoff in Japan (Line 240-241). Additionally, it is difficult using this cutoff value in clinical setting. If there are two infectious patients with eGFR of 40 and 50, the cutoff value will be 1500 and 500, respectively. Please show the reasons why the assessments of presepsin levels were different between definition of sepsis, Sep-1, Sep-C and Sep-3, in the Discussion.

Response

We agree with your comments. We also believe that it is not possible to determine the cut-off for patients with renal impairment based on the results of this study. In order to determine the cut-off, we think that a prospective and large-scale study on the diagnosis of sepsis by Sep-3, which is the diagnostic standard currently being proposed, is necessary. We intend to show the cutoff values obtained from the results of this study(in lines 243–248, 271-272 and 284-285).

Finally, we divided the degree of renal dysfunction into groups with eGFR ≥60 and eGFR <60. The reasons were as follows: 1) eGFR45 and 50 patients have different cut-offs, 2) when separated by eGFR60, the differences in patient number and sepsis frequency are also small, ３) eGFR60 is used in the concept of chronic kidney diseases and is widely accepted as a delimiter for renal dysfunction. We added sentence in lines 165–170.

We described why the assessments of presepsin levels were different between the definitions of sepsis, Sep-1, Sep-C, and Sep-3, in the Discussion as follows(in lines 207–218):

“Sep-1 was proposed after repeated revisions based on the old diagnostic criteria for infectious diseases with SIRS. For this reason, we speculate that the diagnostic criteria for Sep-1 are questionable, resulting in inferior diagnostic accuracy of presepsin. Sep-C was stipulated that there is a diagnosis of sepsis in the medical record or summary at the time of discharge. There are no clear criteria for this diagnosis, but Pre values are expected to play a role. In other words, it is possible that the AUC was high in patients with eGFR <60 because Pre was high in this group and it was easy for doctors to diagnose sepsis. Sep-3 is a newly proposed diagnostic criterion that has not yet been used in the studies regarding Pre; thus, we analyzed this criterion. Because the three diagnostic criteria for sepsis are significantly different, the diagnostic accuracy of Pre and the levels of Pre in the groups with and without sepsis may also differ.”

Minor points

Please describe the detail of the definition of Sep-C.

Response

Thank you for your indication. We apologize for our poor explanation. Sep-C signified that we retrospectively investigated the medical records and that the doctor had diagnosed sepsis. Sep-C could be considered for all subjects; however, problems arise because the diagnostic criteria of each doctor are not clear and the presepsin value itself is expected to affect the diagnosis. We added a description of Sep-C (in lines 80–82 and 210–214).

The description of G1, G2, G3a, G3b, G4 and G5 are inappropriate, because these are used for CKD patients. Probably, there were many patients with AKI or acute exacerbation of CKD. I recommend using ≥90, ≥60 to <90, ≥45 to <60, ≥30 to <45, ≥15 to <30, <15, and dialyzed patients.

Response

We agree with your indication. The subjects are expected to include not only CKD but also AKI patients. We changed the representation of the renal function stage classification to eGFR (mL/min/1.73 m²): ≥90, 60–90, 45–60, 30–45, 15–30, <15, and hemodialyzed patients(HD).

Please describe presepsin levels as median and range or median and interquartile range (IQR). The correlation between presepsin and eGFR should be assessed by non-parametric Spearman's correlation test for two reasons. First, it is unclear whether log presepsin was normally distributed. Second, eGFR seems to be not-normally distributed because eGFR at baseline was reported to be 65.1 ± 37.0.

Response

Thank you for your suggestions. As you pointed out, presepsin values show a non-normal distribution and are thus depicted as median and interquartile range (IQR). We analyzed the relationship between presepsin and eGFR using the non-parametric Spearman's correlation test. Furthermore, for easier reader comprehension, presepsin values were shown according to the eGFR category and compared between patients with and without sepsis. Then, comparisons were made using a non-parametric method.

Table 1 should be presented both whole patients and by presepsin levels, the exposure of interest. It will make easier to understand the clinical characteristics of the patients with higher (or lower) presepsin levels.

Response

Thank you for your advice. We have now provided a patient background divided into those over and below the median serum presepsin level (433 pg/mL) in addition to all subjects (Table 1). In addition, we added the text related to Table 1 (in lines 129–137).

Response to Reviewers #3

Major criticism

1. eGFR was calculated from serum creatinine in patients with acute kidney injury (AKI) or with acute kidney disease (AKD). Serum creatinine is not consistent in the condition of AKI and AKD. In addition, the increasing serum creatinine is delayed from falling true GFR in developing to AKI, while this could be happen vice versa in recovering from AKI. Thus, in the beginning of sepsis, the calculated eGFR could be often higher than true measured GFR, leading to overestimation of true GFR and in the recovering from sepsis, eGFR could be lower than measured GFR. The authors should mention this point in limitation.

Response

Thank you for your advice. Certainly, there may be a discrepancy between eGFRcr and GFR. Unfortunately, we did not collect data on other renal function indicators, such as cystatin C or inulin clearance. However, it seems acceptable to adopt eGFRcr, which is the most used index of renal function in Japanese clinical practice, as a substitute for GFR. We have added the following text (in lines 272–275):

“Second, we used eGFR as an index of renal function, and thus it is possible that AKI cases associated with sepsis were not excluded. Additionally, the time course of sepsis was unknown, which prevented an accurate evaluation of the GFR.”

2. In Fig. 1, some participants were overestimated GFR whose eGFR>200mL/min/1.73m2. I feel these participants might lose muscle mass. I suggest these participants should be excluded from this study.

Response

Thank you for your advice. Certainly, some of the subjects had extremely high eGFRs. It is expected that some of these individuals have low muscle mass and overestimated eGFRs. First, we excluded seven patients with extremely high eGFR (≥200) and one pregnant woman. The top 2.5% of the eGFR distribution in all patients was 139.4 pg/mL, and thus we investigated 13 patients with ≥140 pg/mL and excluded nine patients with a body mass index of <20. This has now been described in the Methods (in lines 54–59).

3. In Fig. 3, approximately 50% of participants were not able to diagnose sepsis or not in each CKD category because of lack of data. I think Sep-3 criteria should not be used for diagnosis of sepsis in this study.

Response

Thank you for your advice. Originally, we wanted to confirm the accuracy of presepsin for the currently proposed diagnosis of Sep-3. However, as a result of retrospective data collection, the main subjects were those who did not have the data necessary to calculate the qSOFA and SOFA scores required for the diagnosis of Sep-3. However, as a result of re-analyzing the diagnosis of Sep-3, 721 cases (86% of the total) could be diagnosed with Sep-3. Sep-1 had a history of revision due to problems with diagnostic accuracy, and Sep-C is expected to have influenced the diagnosis by the presepsin value itself. Thus, we believe that the analyses for Sep-3 should remain.

4. The authors concluded that the optimal cutoff values for presepsin in patients with renal impairment should be approximately 500 pg/mL for ⪯G3a and 1500 pg/mL for ⪰G3b. I think this conclusion solely comes from the results of Sep-C. In addition, these cutoff values might be for only Japanese because this study was conducted only Japanese patients.

Response

We strongly agree with your point. We have now added that this cut-off is a value obtained only from a single-center retrospective study on the Japanese population, and that a future prospective study is necessary to confirm the cut-off (in lines 243–244 and 284–285). The patient number, eGFR, and Sep-3 frequency changed after reanalysis, and the AUC did not change significantly between eGFR <60 and ≥60 only with Sep-3 criteria. For this reason, we set the cut-off based on the results of the analysis against the Sep-3 criterion.

Minor criticism

1. The manuscript contains Figure legends in the main text. Figure legends should be separately noted.

Response

Thank you for your suggestion. I emailed the PLOS ONE editor for clarification on where to place Figure Legends, and I was instructed to list them before the references in the main text. Accordingly, I followed their instructions.

2. The authors showed LogPre differed significantly between patients with and without sepsis. I think the authors also should show the serum presepsin values differ between patients with and without sepsis.

Response

Thank you for your suggestion. As presepsin shows a non-normal and wide distribution, we also considered the appearance of the graph and used LogPre for analysis. The value that converted the value of LogPre into an antilogarithm is shown. However, LogPre does not show a completely normal distribution. Additionally, we showed the value of presepsin with the median and interquartile range (IQR) and changed the results of the analysis with a non-parametric method.
